# Application of Central Upwind Scheme for Solving Special Relativistic Hydrodynamic Equations

**DOI:** 10.1371/journal.pone.0128698

**Published:** 2015-06-12

**Authors:** Muhammad Yousaf, Tayabia Ghaffar, Shamsul Qamar

**Affiliations:** 1 Department of Mathematics, COMSATS Institute of Information Technology, Park Road, Chak Shahzad, Islamabad, Pakistan; 2 Department of Mathematics, Quaid-I-Azam University, Islamabad, Pakistan; University of Aveiro, PORTUGAL

## Abstract

The accurate modeling of various features in high energy astrophysical scenarios requires the solution of the Einstein equations together with those of special relativistic hydrodynamics (SRHD). Such models are more complicated than the non-relativistic ones due to the nonlinear relations between the conserved and state variables. A high-resolution shock-capturing central upwind scheme is implemented to solve the given set of equations. The proposed technique uses the precise information of local propagation speeds to avoid the excessive numerical diffusion. The second order accuracy of the scheme is obtained with the use of MUSCL-type initial reconstruction and Runge-Kutta time stepping method. After a discussion of the equations solved and of the techniques employed, a series of one and two-dimensional test problems are carried out. To validate the method and assess its accuracy, the staggered central and the kinetic flux-vector splitting schemes are also applied to the same model. The scheme is robust and efficient. Its results are comparable to those obtained from the sophisticated algorithms, even in the case of highly relativistic two-dimensional test problems.

## Introduction

The special relativistic hydrodynamical models can be used to simulate many high energy phenomena in astrophysics, including accretion flows, gamma-ray bursts, and jet flows [1, 2]. Moreover, free-electron laser technology, high energy particles beams and heavy-ion collisions can be modeled by using special relativistic hydrodynamics (SRHD) [3]. Since last decade, several upwind high-resolution shock-capturing (HRSC) schemes were being applied to solve relativistic hydrodynamical models. The schemes have high order accuracy in smooth regions of the simulated flow and resolve sharp discontinuous profiles in the shock regions [1, 4–9]. A part from the kinetic scheme which is based on kinetic equation and equilibrium distribution function [10–14], all these techniques are based on the macroscopic continuum description. Moreover, Qamar and Yousaf have implemented the space-time CESE method [15, 16] and discontinuous Galerkin finite element method [17] for solving these RHD equations.

Central schemes could serve as a common numerical technique to solve several scientific and engineering problems due to avoiding the specific eigenstructure of the problem [18]. The schemes have been successfully applied to solve problems in computational fluid dynamics, astrophysics, metrology, semiconductors, shallow flow and multi-component flows [13, 19]. The first-order LaxFriedrichs scheme is the the back bone of such schemes. The central NessyahuTadmor (NT) scheme [20] is a Riemann-solver-free high resolution staggered central scheme which do not need any information about the eignstructure of the problem. Thus, the method can be implemented as a black box solver to any system of conservation laws. However, this family of central schemes suffers from excessive numerical viscosity when a sufficiently small time step is imposed, e.g., due to the presence of degenerate diffusion terms. To overcome this deficiency, Kurganov and Tadmor [18, 21] improved the NT scheme by using the correct information of local propagation speeds and obtained the semi-discrete central upwind scheme. Similar to the staggered NT scheme, it enjoys the benefits of high resolution, simplicity and robustness. However, the central upwind scheme reduces large amount of numerical dissipation present in the NT central schemes. This scheme has already been applied to different problems namely, two-layer shallow water equations [22] and Hamilton Jacobi equations [23].

In this article, the central upwind scheme is implemented for solving the SRHD equations in one and two space dimensions. For validation and comparison, the numerical results of central(NT) and KFVS schemes are also presented [20, 24]. Several numerical cases studies are carried out. It was found that central upwind scheme gives comparable solutions to the KFVS scheme and is superior over the central NT scheme.

The present paper is organized as follows. Section 2 gives a brief description of SRHD equations. The one-dimensional central upwind scheme is briefly presented in Section 3. Afterwards, the scheme is extended to two-space dimensions. In Section 4, one and two-dimensional numerical test problems are given. Conclusions and remarks are offered in Section 5.

## 1 Description of Special Relativistic Hydrodynamics

In this section, we give a general technical description of the SRHD equation as they will be used in the analytic description of different problems and development of our numerical code.

The SRHD equation are obtained from the local conservation laws of the stress-energy tensor *T* ^*μν*^ and the mass flux vector *N* ^*μ*^

$$\begin{array}{c} \frac{\partial N^{\mu }}{\partial x^{\mu }}=0\;\text{and}\;\frac{\partial T^{\mu \nu }}{\partial x^{\mu }}=0,\;\; \end{array}$$ (1)

as $\frac{\partial }{\partial x^{\mu }}$ denotes for the covariant derivative associating with the four space-time coordinates *x* ^*μ*^ = (*t*, *x* ^*i*^). Throughout Greek indices (e.g., *μ*, *ν*) represent the space-time components while Latin indices run from 1 to 3 and units in which *c* (the speed of light) = 1 are used. The components of *N* ^*μ*^ and *T* ^*μν*^ on a coordinate basis are

$$\begin{array}{c} N^{\mu }=\rho u^{\mu }\;\text{and}\;T^{\mu \nu }=\left(e+p\right)u^{\mu }u^{\nu }-pg^{\mu \nu }\;, \end{array}$$ (2)

where, *u* ^*μ*^ representing the 4-velocity of fluid and *ρ* the rest-mass density, *p* is the pressure in a locally inertial reference frame and *g*_*μν*_ = diag(1, −1, −1, −1) is the metric tensor in Minkowski Space. The system composed by the continuity equation and the equation of motion must be supplemented by an equation of state relating the pressure, e.g.

$$\begin{array}{c} e=\rho +\frac{p}{\Gamma -1}\;, \end{array}$$ (3)

where, Γ is adiabatic index with $\Gamma =\frac{5}{3}$ in the case of mildly relativistic.

In Minkowski Spacetime and cartesian coordinates, the local conservation laws describing the motion of a relativistic fluid can be cast as a first-order flux-conservative system of the form

$$\begin{array}{c} \frac{\partial W}{\partial t}+\frac{\partial F(W)}{\partial x}+\frac{\partial G(W)}{\partial y}=0\;, \end{array}$$ (4)

by introducing the conservative variables **W** = (*W*_1_, *W*_2_, *W*_3_, *W*_4_)^*T*^ and the fluxes **F** and **G** are given by

$$\begin{array}{ccc} W(w) & = & {\left(\rho \gamma ,\left(e+p\right)\gamma^{2}v^{1},\left(e+p\right)\gamma^{2}v^{2},\left(e+p\right)\gamma^{2}-p\right)}^{T}\;, \end{array}$$ (5)

$$\begin{array}{ccc} F(W) & = & {\left(\rho \gamma v^{1},\left(e+p\right)\gamma^{2}{\left(v^{1}\right)}^{2}+p,\left(e+p\right)\gamma^{2}v^{1}v^{2},\left(e+p\right)\gamma^{2}v^{1}\right)}^{T}\;,\\ G(W) & = & {\left(\rho \gamma v^{2},\left(e+p\right)\gamma^{2}v^{1}v^{2},\left(e+p\right)\gamma^{2}{\left(v^{2}\right)}^{2}+p,\left(e+p\right)\gamma^{2}v^{2}\right)}^{T}, \end{array}$$ (6)

where, *v* ^*i*^ is the 3-velocity and *γ* is the Lorentz factor satisfying $\gamma =u^{0}=\frac{1}{\sqrt{1-{\left(v\right)}^{2}}}$ with (*v*)^2^ = (*v* ^1^)^2^ + (*v* ^2^)^2^ + (*v* ^3^)^2^. The 3-velocity components are obtained from the spatial components of the 4-velocity as $v^{i}=\frac{u^{i}}{u^{0}}$ (normalized as *u* ^*μ*^ *u*_*μ*_ = 1) and the conservative variables are related with physical or primitive variables, **w** = (*ρ*, *v* ^1^, *v* ^2^, *p*)^*T*^.

### 1.1 Recovery of physical variables

We need to convert conservative variables to physical variables. Here, *W*_*m*_, *m* = 1, 2, 3, 4, will denote the conservative variables while

$$\begin{array}{c} w_{1}=\rho ,\;w_{i+1}=v^{i},\;i=1,2,\;\text{and}\;w_{4}=p\; \end{array}$$ (7)

are the physical variables. From Eqs (5)–(7), it is concluded that each *W*_*m*_ and each flux functions are the functions of the physical variables. To show that each physical variable is an implicit function of the conservative variables *W*_*m*_, let

$$\begin{array}{c} D=W_{1},\;Q^{i}=W_{i+1},\;i=1,2,\;\text{and}\;E=W_{4}\; \end{array}$$ (8)

and

$$\begin{array}{c} U=\left(e+p\right)\gamma^{2}\;\text{and}\;\Gamma_{1}=\Gamma /\left(\Gamma -1\right)\;. \end{array}$$ (9)

Then with the help of Eqs (3) and (5), we get

$$\begin{array}{c} U=\left(\rho +\Gamma_{1}p\right)\gamma^{2}=D\gamma +\Gamma_{1}\gamma^{2}p\; \end{array}$$ (10)

and

$$\begin{array}{c} E=W_{4}=U-p\;. \end{array}$$ (11)

By eliminating the parameter *p* from Eqs (10) and (11), one has

$$\begin{array}{c} U=U\left(D,E,\gamma \right)=\frac{E\Gamma_{1}\gamma^{2}-D\gamma }{\Gamma_{1}\gamma^{2}-1}\;\left(\Gamma_{1}\gamma^{2}-1\neq 0\right)\;. \end{array}$$ (12)

Moreover, by using Eqs (5), (8), (9) and (12), we have

$$\begin{array}{c} {\left(v\right)}^{2}=1-\frac{1}{\gamma^{2}}\; \end{array}$$ (13)

and

$$\begin{array}{ccc} {\left(Q\right)}^{2} & = & \sum_{i=1}^{2}{\left(Q^{i}\right)}^{2}=\sum_{i=1}^{2}{\left(W_{i+1}\right)}^{2}={\left[\left(e+p\right)\gamma^{2}\right]}^{2}\sum_{i=1}^{2}{\left(v^{i}\right)}^{2}\\ & = & {\left[U(D,E,\gamma )\right]}^{2}{\left(v\right)}^{2}={\left[U(D,E,\gamma )\right]}^{2}\left(1-\gamma^{-2}\right)\;. \end{array}$$ (14)

Thus

$$\begin{array}{c} {\left[U(D,E,\gamma )\right]}^{2}\left(1-\gamma^{-2}\right)-{\left(Q\right)}^{2}=0\;. \end{array}$$ (15)

For the solution of nonlinear Eq (15), the Newton routine was employed to find its root *γ*. In our numerical computations, four to six iterations were required to obtain a root of tolerance 10^−6^. According to Eqs (14) and (15), the parameter *γ* is an implicit function of *D*, *E* and (*Q*)^2^ in *W*_*m*_, *m* = 1, 2, 3, 4. Moreover, by using direct results from Eqs (5), (8), (9) and (11), i.e.,

$$\begin{array}{c} \rho =\frac{D}{\gamma }\;,\;v^{i}=\frac{Q^{i}}{U}\;,\;m=1,2,\;\;\text{and}\;p=U-E. \end{array}$$ (16)

It is seen that the physical variables are implicit functions of the conservative variables.

## 2 Numerical Schemes

### 2.1 One-dimensional central upwind scheme

In this section, the semi discrete central-upwind scheme is derived [18]. The above SRHD model in one space dimension can be expressed as

$$\begin{array}{c} W_{t}+F{\left(W\right)}_{x}=0\;. \end{array}$$ (17)

Before applying the scheme, it is necessary to discretize the computational domain. Let *N* denote the number of discretization points and ${\left(x_{i-\frac{1}{2}}\right)}_{i\in \{1,\cdots ,N+1\}}$ are partitions of the given interval [0, *x*_max_]. For each *i* = 1, 2, ⋯, *N*, Δ*x* is a constant width of each mesh interval, *x*_*i*_ denote the cell centers, and $x_{i\pm \frac{1}{2}}$ refer as cell boundaries. We assign,

$$\begin{array}{c} x_{1/2}=0\;,\;x_{N+1/2}=x_{\max}\;,\;x_{i+1/2}=i\cdot \Delta x\;,\;\;\;\;\text{for}\;\;\;i=1,2,\cdots N\;. \end{array}$$ (18)

Moreover,

$$\begin{array}{c} x_{i}=\left(x_{i-1/2}+x_{i+1/2}\right)/2\;\;\text{and}\;\Delta x=x_{i+1/2}-x_{i-1/2}=\frac{x_{\max}}{N+1}\;. \end{array}$$ (19)

Let Ω_*i*_: = [*x*_*i*−1/2_, *x*_*i*+1/2_] for *i* ≥ 1. In each Ω_*i*_, the averaged values of the conserved variable **W**(*t*) are defined as

$$\begin{array}{c} W_{i}≔W_{i}\left(t\right)=\frac{1}{\Delta x}\int_{\Omega_{i}}W\left(x,t\right)\;dx\;. \end{array}$$ (20)

By integrating Eq (17) over the interval Ω_*i*_ = [*x*_*i*−1/2_, *x*_*i*+1/2_], we obtain

$$\begin{array}{c} \frac{dW_{i}}{dt}=-\frac{S_{i+\frac{1}{2}}\left(t\right)-S_{i-\frac{1}{2}}\left(t\right)}{\Delta x}. \end{array}$$ (21)

The numerical fluxes are defined as

$$\begin{array}{c} S_{i+\frac{1}{2}}=\frac{F\left(W_{i+\frac{1}{2}}^{+}\right)+F\left(W_{i+\frac{1}{2}}^{-}\right)}{2}-\frac{a_{i+\frac{1}{2}}}{2}(W_{i+\frac{1}{2}}^{+}-W_{i+\frac{1}{2}}^{-})\;, \end{array}$$ (22)

Here, **W** ^+^ and **W** ^−^ are the point values of the piecewise linear reconstruction $\tilde{\text{W}}=(\tilde{\rho },\tilde{\rho u},\tilde{E^{*}})$ for **W**, namely

$$\begin{array}{c} W_{i+\frac{1}{2}}^{+}=W_{i+1}-\frac{\Delta x}{2}W_{i+1}^{x},\;W_{i+\frac{1}{2}}^{-}=W_{i}+\frac{\Delta x}{2}W_{i}^{x}. \end{array}$$ (23)

The numerical derivatives ${\text{W}}_{i}^{x}$ are at least first-order approximations of **W**_*x*_(*x*_*i*_, *t*) and are computed using a nonlinear limiter that would ensure the non-oscillatory nature of reconstruction (23). A possible computation of these slopes is given by family of discrete derivatives parameterized with 1 ≤ *θ* ≤ 2, for example

$$\begin{array}{c} W_{i}^{x}=MM\;\{\theta \Delta W_{i+\frac{1}{2}},\frac{\theta }{2}\;(\Delta W_{i+\frac{1}{2}}+\Delta W_{i-\frac{1}{2}}),\theta \Delta W_{i-\frac{1}{2}}\}\;, \end{array}$$ (24)

$$\begin{array}{c} \Delta W_{i+\frac{1}{2}}=W_{i+1}-W_{i}\;. \end{array}$$

$$\begin{array}{c} MM\left\{x_{1},x_{2},...\right\}=\{\begin{array}{c} {\text{min}}_{i}\left\{x_{i}\right\}\;\text{if}\;\;x_{i}>0\;\forall i\;,\\ {\text{max}}_{i}\left\{x_{i}\right\}\;\text{if}\;\;x_{i}<0\;\forall i\;,\\ 0\;\;\text{otherwise}\;. \end{array} \end{array}$$ (25)

Where, Δ denotes central differencing and *MM* is the min-mod nonlinear limiter. Further, the local one sided speed at $x_{i+\frac{1}{2}}$ is given as

$$\begin{array}{c} a_{i+\frac{1}{2}}\left(t\right)=\max\;\{\rho \;(\frac{\partial F}{\partial W}\left(W_{i+\frac{1}{2}}^{+}\left(t\right)\right)),\rho \;(\frac{\partial F}{\partial W}\left(W_{i+\frac{1}{2}}^{-}\left(t\right)\right))\}. \end{array}$$ (26)

To obtain second order accuracy in time, we use a second order TVD Runge-Kutta scheme to solve Eq (21). Denoting the right-hand side of Eq (21) as *L*(**W**), a second order TVD Runge-Kutta scheme updates **W** through the following two stages

$$\begin{array}{c} W^{\left(1\right)}=W^{n}+\Delta tL\left(W^{n}\right)\;, \end{array}$$ (27a)

$$\begin{array}{c} W^{n+1}=\frac{1}{2}\;(W^{n}+W^{\left(1\right)}+\Delta t\;L\left(W^{\left(1\right)}\right))\;, \end{array}$$ (27b)

where, **W** ^*n*^ is a solution at previous time step *t* ^*n*^ and **W** ^*n*+1^ is updated solution at next time step *t* ^*n*+1^. Moreover, Δ*t* represents the time step.

### 2.2 One-dimensional central scheme

We briefly give the high-resolution non-oscillatory central schemes of Nessyahu and Tadmor [20] for the solution of current SRHD model. These are the predictor-corrector type methods consist of two steps. In the predictor step, we use the non-oscillatory piecewise-linear reconstructions of the cell averages to predict the midpoint values. In the corrector step, staggered averaging along with the predicted mid-values are employed to get the updated cell averaged solution. The scheme can be summarized as

$$\begin{array}{c} \text{Predictor:}\;W_{i}^{n+\frac{1}{2}}=W_{i}^{n}-\frac{\xi }{2}F^{x}\left(W_{i}^{n}\right)\;, \end{array}$$ (28)

$$\begin{array}{ccc} \text{Corrector:}\;W_{i+\frac{1}{2}}^{n+1} & = & \frac{1}{2}\left(W_{i}^{n}+W_{i+1}^{n}\right)+\frac{1}{8}\left(W_{i}^{x}-W_{i+1}^{x}\right)\\ & & -\xi \;[F_{i+1}^{n+\frac{1}{2}}-F_{i}^{n+\frac{1}{2}}]\;, \end{array}$$ (29)

where, *ξ* = Δ*t*/Δ*x*. Moreover, $\frac{1}{\Delta x}{\text{F}}^{x}({\text{W}}_{i})$ stands for an approximate numerical derivatives of the flux **F**(*t*, *x* = *x*_*i*_)

$$\begin{array}{c} \frac{1}{\Delta x}F^{x}\left(W_{i}\right)=\frac{\partial }{\partial x}F(w\left(t,x=x_{i}\right)+O\left(\Delta x\right)\;. \end{array}$$ (30)

The fluxes **F** ^*x*^(**W**_*i*_) are computed by the same manner as for **W** ^*x*^.

### 2.3 Two-dimensional central upwind scheme

Consider the two-dimensional SRHD equation (c.f. Eq (4))

$$\begin{array}{c} W_{t}+F{\left(W\right)}_{x}+G{\left(W\right)}_{y}=0\;, \end{array}$$ (31)

Let *N*_*x*_ and *N*_*y*_ denote large integers in the *x* and *y*–directions, respectively. We assume a Cartesian mesh with a rectangular domain [*x*_0_, *x*_max_] × [*y*_0_, *y*_max_] which is covered by cells $C_{ij}\equiv \left[x_{i-\frac{1}{2}},x_{i+\frac{1}{2}}\right]\times \left[y_{j-\frac{1}{2}},y_{j+\frac{1}{2}}\right]$ for 1 ≤ *i* ≤ *N*_*x*_ and 1 ≤ *j* ≤ *N*_*y*_. The representative coordinates of the population in cell *C*_*ij*_ are represented by (*x*_*i*_, *y*_*j*_). Here,

$$\begin{array}{c} \left(x_{1/2},x_{1/2}\right)=\left(0,0\right),\;\;x_{i}=\frac{x_{i-1/2}+x_{i+1/2}}{2},\;\;y_{j}=\frac{y_{j-1/2}+y_{j+1/2}}{2}, \end{array}$$ (32)

and

$$\begin{array}{c} \;\Delta x_{i}=x_{i+1/2}-x_{i-1/2}\;,\;\Delta y_{j}=y_{j+1/2}-y_{j-1/2}\;. \end{array}$$ (33)

The cell averaged values of conserved variables **W**_*i*,*j*_(*t*) at any time *t* is

$$\begin{array}{c} W_{i,j}≔W_{i,j}\left(t\right)=\frac{1}{\Delta x_{i}\Delta y_{j}}\int_{C_{ij}}W\left(x,y,t\right)\;dydx\;. \end{array}$$ (34)

and the piecewise linear interplant is

$$\begin{array}{c} W\left(x,y,t\right)=\sum_{i,j}[W_{i,j}+{\left(W^{x}\right)}_{i,j}\left(x-x_{i}\right)+{\left(W^{y}\right)}_{i,j}\left(y-y_{j}\right)]\chi_{i,j}, \end{array}$$ (35)

where, *χ*_*i*,*j*_ is the characteristic function for the corresponding cell $\left(x_{i-\frac{1}{2}},x_{i+\frac{1}{2}})\times (y_{j-\frac{1}{2}},y_{j+\frac{1}{2}}\right)$, (**W** ^*x*^)_*i*,*j*_ and (**W** ^*y*^)_*i*,*j*_ are the approximations of the *x*- and *y*-derivatives of **W** at the cell centers (*x*_*i*_, *y*_*j*_). The generalized MM limiter is used for the computation of these partial derivatives to avoid oscillations

$$\begin{array}{ccc} {\left(W^{x}\right)}_{i,j}^{n} & = & MM\;(\theta \frac{W_{i+1,j}-W_{i,j}}{\Delta x},\frac{W_{i+1,j}-W_{i-1,j}}{2\Delta x},\theta \frac{W_{i,j}-W_{i-1,j}}{\Delta x})\;\\ {\left(W^{y}\right)}_{i,j} & = & MM\;(\theta \frac{W_{i,j+1}-W_{i,j}}{\Delta y},\frac{W_{i,j+1}-W_{i,j-1}}{2\Delta y},\theta \frac{W_{i,j}-W_{i,j-1}}{\Delta y}), \end{array}$$ (36)

where, 1 ≤ *θ* ≤ 2 and *MM* is defined above. After integrating the two-dimensional SRHD Eq (31) over the control volume *C*_*ij*_, the two-dimensional extension of the scheme can be expressed as

$$\begin{array}{c} \frac{dW_{i,j}}{dt}=-\frac{S_{i+\frac{1}{2},j}^{x}-S_{i-\frac{1}{2},j}^{x}}{\Delta x}-\frac{S_{i,j+\frac{1}{2}}^{y}-S_{i,j-\frac{1}{2}}^{y}}{\Delta y}\;. \end{array}$$ (37)

Here,

$$\begin{array}{ccc} S_{i+\frac{1}{2},j}^{x} & = & \frac{F\left(W_{i+\frac{1}{2},j}^{-}\right)+F\left(W_{i+\frac{1}{2},j}^{+}\right)}{2}+\frac{a_{i+\frac{1}{2},j}^{x}}{2}(W_{i+\frac{1}{2},j}^{-}-W_{i+\frac{1}{2},j}^{+}),\;\\ S_{i,j+\frac{1}{2}}^{y} & = & \frac{G\left(W_{i,j+\frac{1}{2}}^{-}\right)+G\left(W_{i,j+\frac{1}{2}}^{+}\right)}{2}+\frac{a_{i,j+\frac{1}{2}}^{y}}{2}(W_{i,j+\frac{1}{2}}^{-}-W_{i,j+\frac{1}{2}}^{+})\;, \end{array}$$ (38)

where, the intermediate values are defined as

$$\begin{array}{ccc} W_{i+\frac{1}{2},j}^{-} & = & W_{i,j}+\frac{\Delta x}{2}{\left(W^{x}\right)}_{i,j}\;,\;W_{i+\frac{1}{2},j}^{+}=W_{i+1,j}-\frac{\Delta x}{2}{\left(W^{x}\right)}_{i+1,j}\\ W_{i,j+\frac{1}{2}}^{-} & = & W_{i,j}+\frac{\Delta y}{2}{\left(W^{y}\right)}_{i,j}\;,\;W_{i,j+\frac{1}{2}}^{+}=W_{i,j+1}-\frac{\Delta y}{2}{\left(W^{y}\right)}_{i,j+1}\;. \end{array}$$ (39)

Since both $a_{i+\frac{1}{2},j}^{x}$ and $a_{i,j+\frac{1}{2}}^{y}$ are the local speeds which can be calculated as

$$\begin{array}{ccc} a_{i+\frac{1}{2},j}^{x} & = & \max_{\pm }\rho \;(\frac{\partial F}{\partial W}\left(W_{i+\frac{1}{2},j}^{\pm }\right)),\;\\ a_{i,j+\frac{1}{2}}^{y} & = & \max_{\pm }\rho \;(\frac{\partial G}{\partial W}\left(W_{i,j+\frac{1}{2}}^{\pm }\right))\;. \end{array}$$ (40)

Complete derivation of the scheme introduced in [18].

### 2.4 Two-dimensional central scheme

This scheme was proposed by Jaing and Tadmor [25]. The scheme has a two-step predictor-corrector type. We start with the cell averages, ${\text{W}}_{i,j}^{n}$, the first-order predictor step is used to get midpoint values, ${\text{W}}_{i,j}^{n+\frac{1}{2}}$, followed by the second-order corrector step to compute the new cell averages ${\text{W}}_{i,j}^{n+1}$. Similar to the 1D case, no exact (approximate) Riemann solver is required. The non-oscillatory property of the scheme depends upon the reconstructed discrete slopes, **W** ^*x*^, **W** ^*y*^, **F** ^*x*^(*w*), and **G** ^*y*^(*w*). At each time step, the grid is staggered to skip the flux calculation at the cell interfaces. The scheme can be written as below.

In the predictor step one has to calculate the mid-point values

$$\begin{array}{c} W_{i,j}^{n+\frac{1}{2}}=W_{i,j}^{n}-\frac{\xi }{2}F^{x}\left(W_{i,j}^{n}\right)-\frac{\eta }{2}G^{y}\left(W_{i,j}^{n}\right)\;, \end{array}$$ (41)

where, *ξ* = Δ*t*/Δ*x* and *η* = Δ*t*/Δ*y*. This step is followed by a corrector step to get the updated values at the next time step

$$\begin{array}{ccc} W_{i+\frac{1}{2},j+\frac{1}{2}}^{n+1} & = & \frac{1}{4}\left(W_{i,j}^{n}+W_{i+1,j}^{n}+W_{i,j+1}^{n}+W_{i+1,j+1}^{n}\right)\\ & & +\frac{1}{16}\left(W_{i,j}^{x}-W_{i+1,j}^{x}\right)-\frac{\xi }{2}\;(F_{i+1,j}^{n+\frac{1}{2}}-F_{i,j}^{n+\frac{1}{2}})\;\\ & & +\frac{1}{16}\left(W_{i,j+1}^{x}-W_{i+1,j+1}^{x}\right)-\frac{\xi }{2}\;(F_{i+1,j+1}^{n+\frac{1}{2}}-F_{i,j+1}^{n+\frac{1}{2}})\;\\ & & +\frac{1}{16}\left(W_{i,j}^{y}-W_{i,j+1}^{y}\right)-\frac{\eta }{2}\;(G_{i,j+1}^{n+\frac{1}{2}}-G_{i,j}^{n+\frac{1}{2}})\;\\ & & +\frac{1}{16}\left(W_{i+1,j}^{y}-W_{i+1,j+1}^{y}\right)-\frac{\eta }{2}\;(G_{i+1,j+1}^{n+\frac{1}{2}}-G_{i+1,j}^{n+\frac{1}{2}})\;. \end{array}$$

This completes the derivation of numerical schemes.

## 3 Numerical Tests

Here, some test problems are presented to validate our numerical scheme.

### 3.1 One-dimensional test problems

In this section, one-dimensional numerical test problems are considered. The results of proposed scheme are validated against those obtained from the central and KFVS schemes [20, 24]. For the considered problems, the computational costs of all schemes were of the order of few seconds.

Before going to the shock tube problems, we analyze the accuracy of schemes for smooth initial data in Problem 1.

#### Problem 1: Experimental order of convergence (EOC)

In this problem, we compare EOCs of the central upwind, the central (NT) and the KFVS methods. The initial data are taken as

$$\begin{array}{ccc} \rho & = & \frac{1}{\sqrt{2\pi }\sigma }\;\;e^{-[\frac{{\left(x-\mu \right)}^{2}}{2\sigma^{2}}]},\;\;\text{with}\;\;\;\sigma =0.13\;\;\text{and}\;\;\;\;\mu =0.5, \end{array}$$ (42)

$$\begin{array}{ccc} v & = & 0.0,\;\;\;p=1.0. \end{array}$$ (43)

The domain is taken to be 0 ≤ *x* ≤ 1, and the final simulation time *t* is 0.5. If *h* = Δ*x* then *L* ^1^-norm is defined as

$$\begin{array}{c} ∥W\left(t,.\right)-W_{h}\left(t,.\right){∥}_{L^{1}\left(\mathbb{R}\right)}=ch^{\alpha }. \end{array}$$ (44)

Here, *α* denotes its order, **W** is the exact solution and **W**_*h*_ is the approximate solution. We define the *L* ^1^-error as $∥\text{W}(t,.)-{\text{W}}_{h}(t,.){∥}_{L^{1}}=\sum_{i=1}^{N}|\text{W}(t,.)-{\text{W}}_{h}(t,.)|\Delta x$, where N is the number of grid elements. Then, Eq (44) gives

$$\begin{array}{c} EOC\overset{\text{def}}{=}\alpha =\ln(\frac{∥W\left(t,.\right)-W_{\frac{h}{2}}\left(t,.\right){∥}_{L^{1}}}{∥W\left(t,.\right)-W_{h}\left(t,.\right){∥}_{L^{1}}})/\ln\left(\frac{1}{2}\right). \end{array}$$

**Fig 1:**
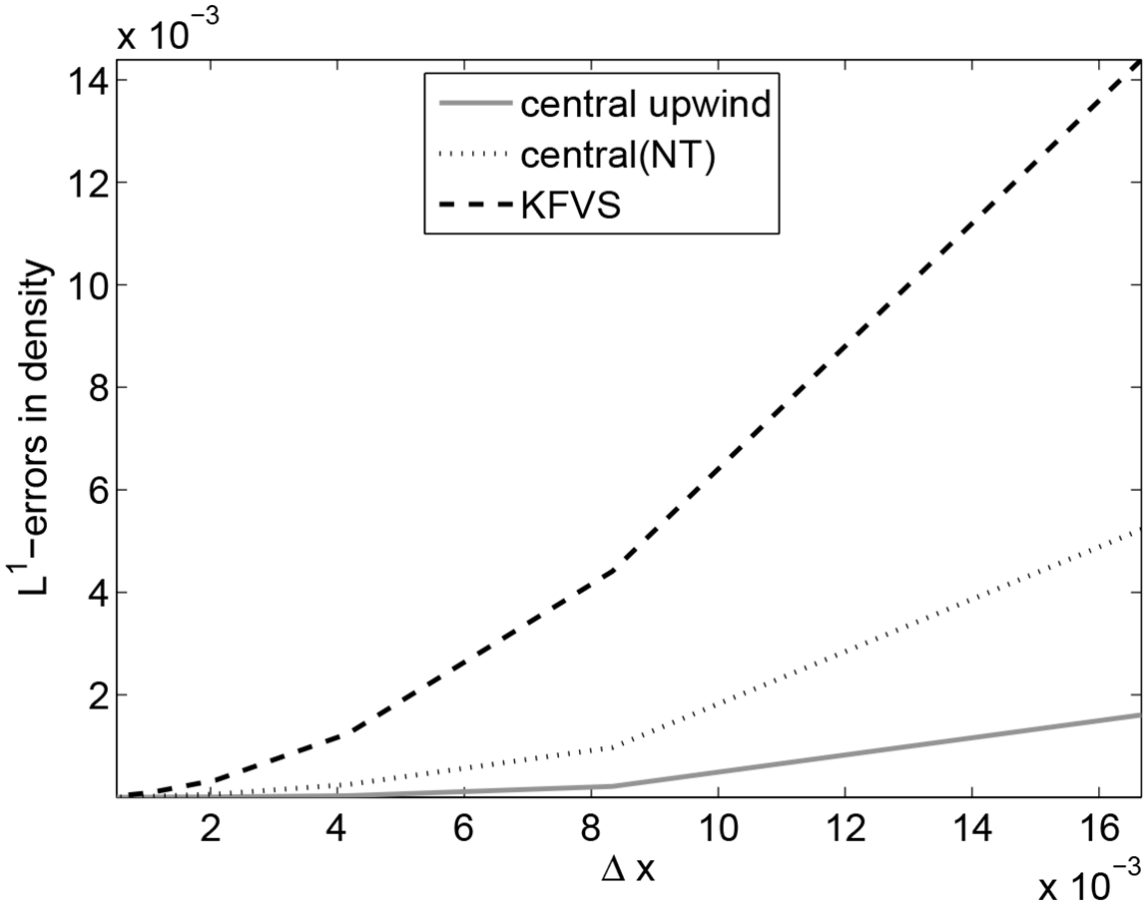
Comparison of *L* ^1^-errors in the schemes.

Table 1 presents *L* ^1^−errors and EOCs of the central upwind, the central (NT) and the KFVS methods. It can be observed that the central upwind scheme produces less errors in the solution and the central scheme has large errors. It is also dipicted from Fig 1. Moreover, all schemes have second order convergence rates.

**Table 1:** Comparison of *L* ^1^-errors and EOC in the schemes.

	Central upwind		Central(NT)		KFVS	
N	*L* ^1^ − *error*	*EOC*	*L* ^1^ − *error*	*EOC*	*L* ^1^ − *error*	*EOC*
60	0.00160781580		0.00522649392		0.01439462580	
120	0.00021363982	2.9118	0.00097061666	2.43	0.00441246472	1.7059
240	0.00003074447	2.7968	0.00024756245	1.97	0.00124106908	1.8300
480	0.00000518274	2.5685	0.00006299962	1.97	0.00033419385	1.8928
960	0.00000112426	2.2047	0.00001851696	1.77	0.00009005563	1.8918
1920	0.00000030025	1.9047	0.00000378190	2.29	0.00002383608	1.9177

#### Shock tube problems

Here, the central upwind scheme is applied to simulate the discontinuous profiles of shock-tube problems. Given a numerical 1D pipe of unit length having *N* grid points, (*ρ*, *v*, *p*) and (*ρ*, *v*, *p*) being the two constant states on the left (0 < *x* ≤ 0.5) and on the right (0.5 < *x* ≤ 1), respectively. These states are with respect to a diaphragm. This diaphragm is placed initially at the middle of the pipe and then pull out. Shocks, contact discontinuities and rarefaction waves are the typical patterns which can be seen in the subsequent evolution. In the relativistic setup, these attributes does not change qualitatively, since the configuration of the characteristics is the same but density jumps could not be limited by a function of the adiabatic index and rarefaction waves do not produce straight profiles due to the nonlinear Lorentz transformation [4]. To validate our proposed central upwind scheme, the results are compared with those of high resolution central(NT) and KFVS methods [20, 24] as well as with the exact Riemann solver [8, 26].

#### Problem 2: Shock tube problem I

The initial data are taken as

$$\begin{array}{ccc} {\left(\rho ,v,p\right)}^{L} & = & (10.0,0.0,13.33)\;,\\ {\left(\rho ,v,p\right)}^{R} & = & (1.0,0.0,0.66\times 10^{-6}). \end{array}$$

**Fig 2:**
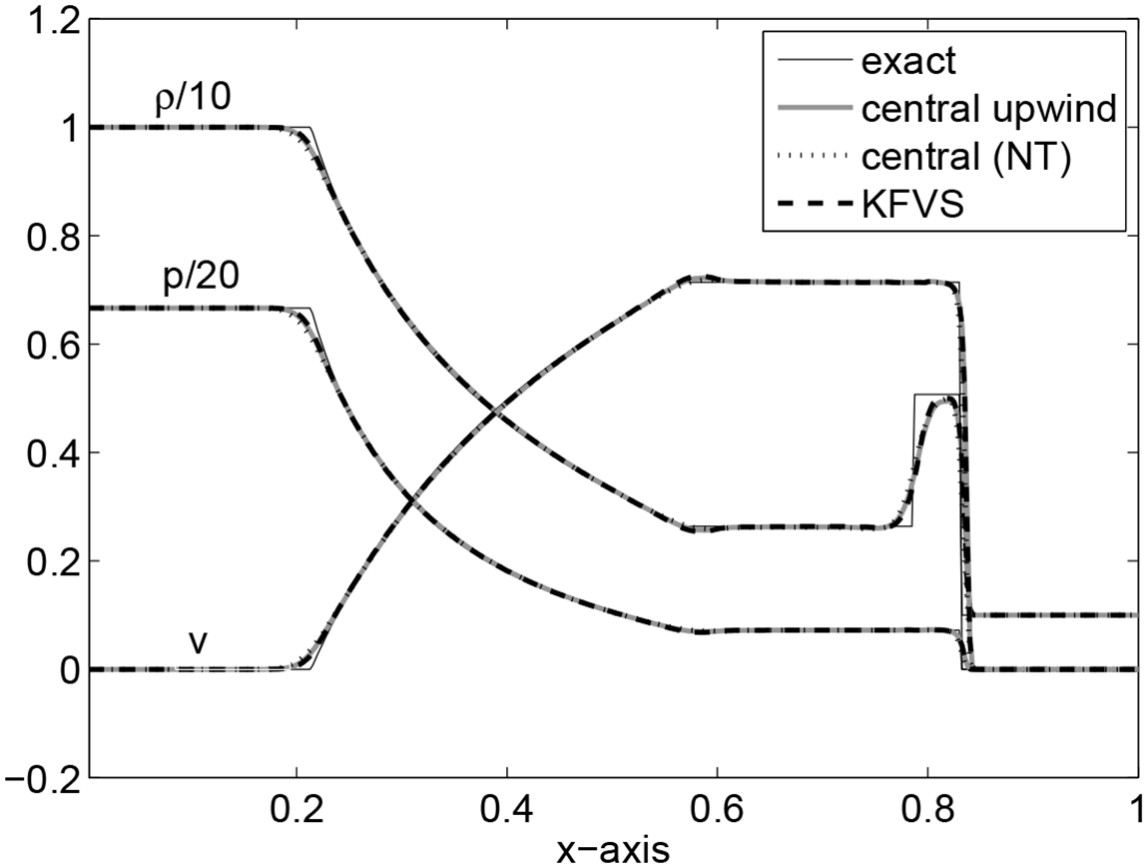
Results of Shock tube problem I at time *t* = 0.4.

The computational domain is taken to be [0, 1] which is discretized into 400 grid points and *t* = 0.4 is the final simulation time. This problem is concerned with the formation of an intermediate state which is bounded by a transonic rarefaction wave propagating towards left and a shock wave propagating towards right. The fluid in this state flows at a mildly relativistic speed (*v* = 0.72*c*) to the right. Fluid particles are accumulated in a dense shell behind the shock wave compressing the fluid and heating it. From a thermodynamic point of view, the fluid is extremely relativistic but mildly relativistic dynamically. Fig 2 shows the results. It can be seen that all three schemes give equivalent results.

#### Problem 3: Shock tube problem II

The initial data are chosen as

$$\begin{array}{ccc} {\left(\rho ,v,p\right)}^{L} & = & (1.0,0.0,1000)\;,\\ {\left(\rho ,v,p\right)}^{R} & = & (1.0,0.0,0.01). \end{array}$$

**Fig 3:**
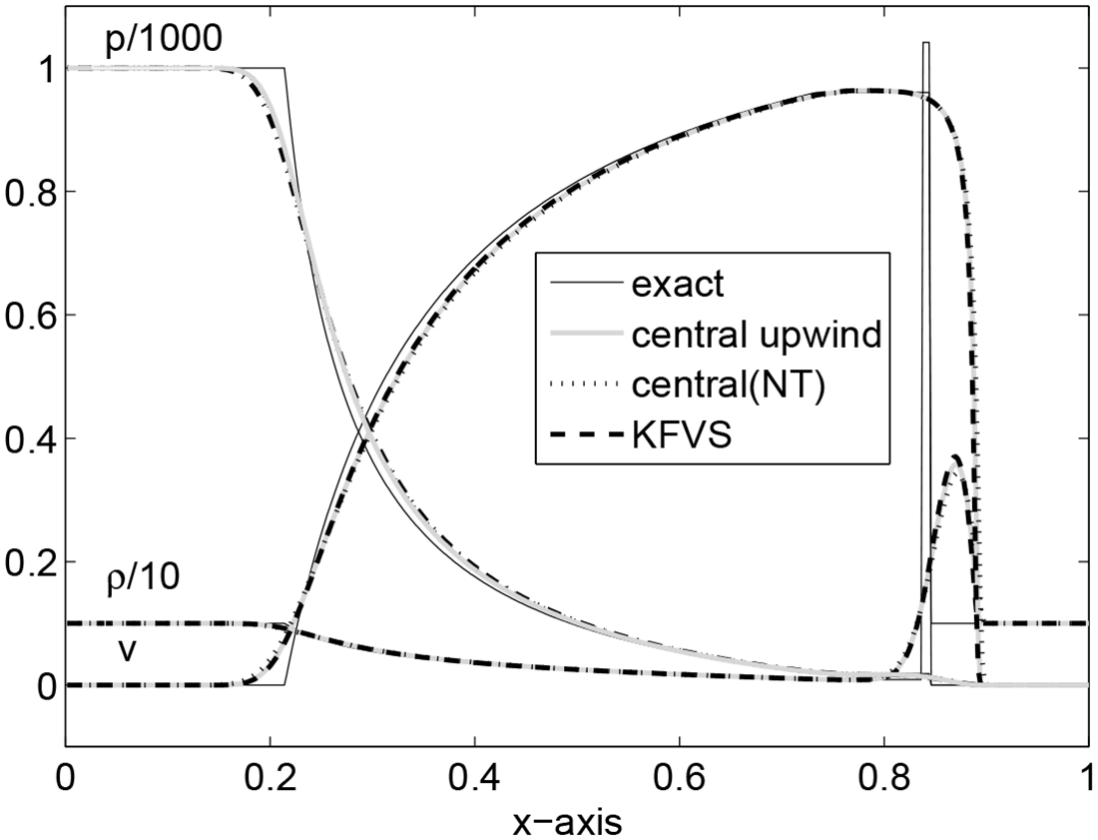
Results of Shock tube problem II at time *t* = 0.35.

The spatial domain is taken as [0, 1] with 500 grid points and *t* = 0.35 is the final time of simulation. The flow pattern is similar to that observed in Problem 2 but is more extreme now. Relativistic effects reduce the post-shock state to a thin dense shell which has width about 1% of the grid size at *t* = 0.35. The fluid velocity is *v* = 0.96*c*. The jump in the density is 10.6 for the exact Riemann solution while 3.9 in the case of the central upwind solution. A similar patterns were observed in [4] by using third order HHL and LLF solvers. The results are given in Fig 3. Here, the results of the central upwind and KFVS schemes seems better than the central(NT) scheme and are equivalent to those available in [4].

#### Problem 4: Perturbed relativistic shock tube flow

**Fig 4:**
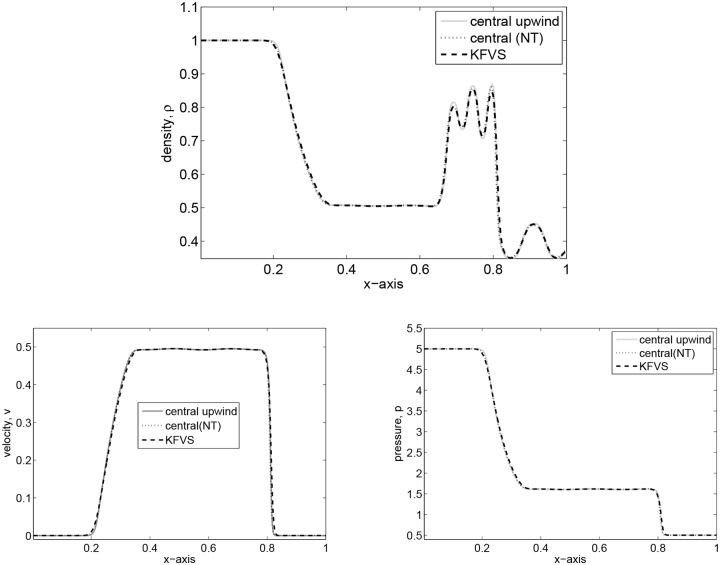
Results of perturbed relativistic shock tube flow at time *t* = 0.35.

This problem was considered by [4, 14]. The conditions at time *t* = 0 are specified as (*ρ*, *v*, *p*)^*L*^ = (5, 0, 50) for 0 ≤ *x* ≤ 0.5 and (*ρ*, *v*, *p*)^*R*^ = (*ρ*_*R*_, 0, 5) for 0.5 ≤ *x* ≤ 1.0. Here, a perturbed density field of a sinusoidal wave, *ρ*_*R*_ = 2 + 0.3 sin(50*x*) is on the right state. The numerical results are obtained on 400 grid elements. The computed solutions are plotted at *t* = 0.35. The results for particle density *ρ*, velocity *v* and pressure *p* are shown in Fig 4. It can be observed that the central upwind scheme captured the sinusoidal profile better than central(NT) and KFVS schemes. Moreover, the results of central upwind scheme are better than those presented in [14].

### 3.2 Two-Dimensional Tests

Here, the 2D test problems are presented. The 2D numerical simulations are more complicated as compared to the 1D case. Here, both components of velocity are interpolated separately and, thus, the condition (*v*)^2^ < 1 may not satisfy in the case of ultra-relativistic due to the effect of numerical errors.

#### Problem 5: EOC in two space dimensions

In this problem, we compare the EOCs of suggested numerical methods in two space dimensions. The initial data are taken as

$$\begin{array}{c} \rho =\frac{1}{\sqrt{2\pi }\sigma }\;\;e^{-[\frac{{\left(x-\mu \right)}^{2}}{2\sigma^{2}}+\frac{{\left(y-\mu \right)}^{2}}{2\sigma^{2}}]},\text{with}\;\;\sigma =0.13\;\;\;\text{and}\;\;\;\;\mu =0.5,\\ v=0,\;\;\;p=1. \end{array}$$ (45)

The domain is chosen to be 0 ≤ *x* ≤ 1, 0 ≤ *y* ≤ 1 and the total simulation time *t* is 0.5. Table 2 gives *L* ^1^−errors and EOCs for the central upwind, the central (NT) and the KFVS methods. It can be observed that the central upwind scheme produces less errors in the solution as compared to the other schemes. Moreover, all schemes have second order convergence rates.

**Table 2:** Comparison of *L* ^1^-errors and EOC in the schemes.

	Central upwind		Central(NT)		KFVS	
N	*L* ^1^ − *error*	*EOC*	*L* ^1^ − *error*	*EOC*	*L* ^1^ − *error*	*EOC*
30	0.000047056586445		0.001251733634641		0.000825512015137	
60	0.000014132555355	1.7354	0.000208094899187	2.59	0.000163604138051	2.3351
120	0.000004568549676	1.6292	0.000044586868191	2.22	0.000038476608572	2.0882
240	0.000001427658873	1.6781	0.000011605047500	1.94	0.000010383848554	1.8896
480	0.000000441460048	1.6933	0.000003428394149	1.76	0.000010383848554	1.7560

#### Problem 6: Two-dimensional shock tube problem

Here, a 2D shock-tube problem is investigated [4]. A square domain of unit side length is taken which is divided into 4 quadrants. The initial conditions are taken as

$$\begin{array}{ccc} {\left(\rho ,v^{1},v^{2},p\right)}^{NE} & = & (1.0,0,0,0.01)\;,\\ {\left(\rho ,v^{1},v^{2},p\right)}^{NW} & = & (1.0,0.99,0,1)\;,\\ {\left(\rho ,v^{1},v^{2},p\right)}^{SW} & = & (5.0,0,0,1)\;,\\ {\left(\rho ,v^{1},v^{2},p\right)}^{SE} & = & (1.0,0,0.99,1)\;. \end{array}$$

**Fig 5:**
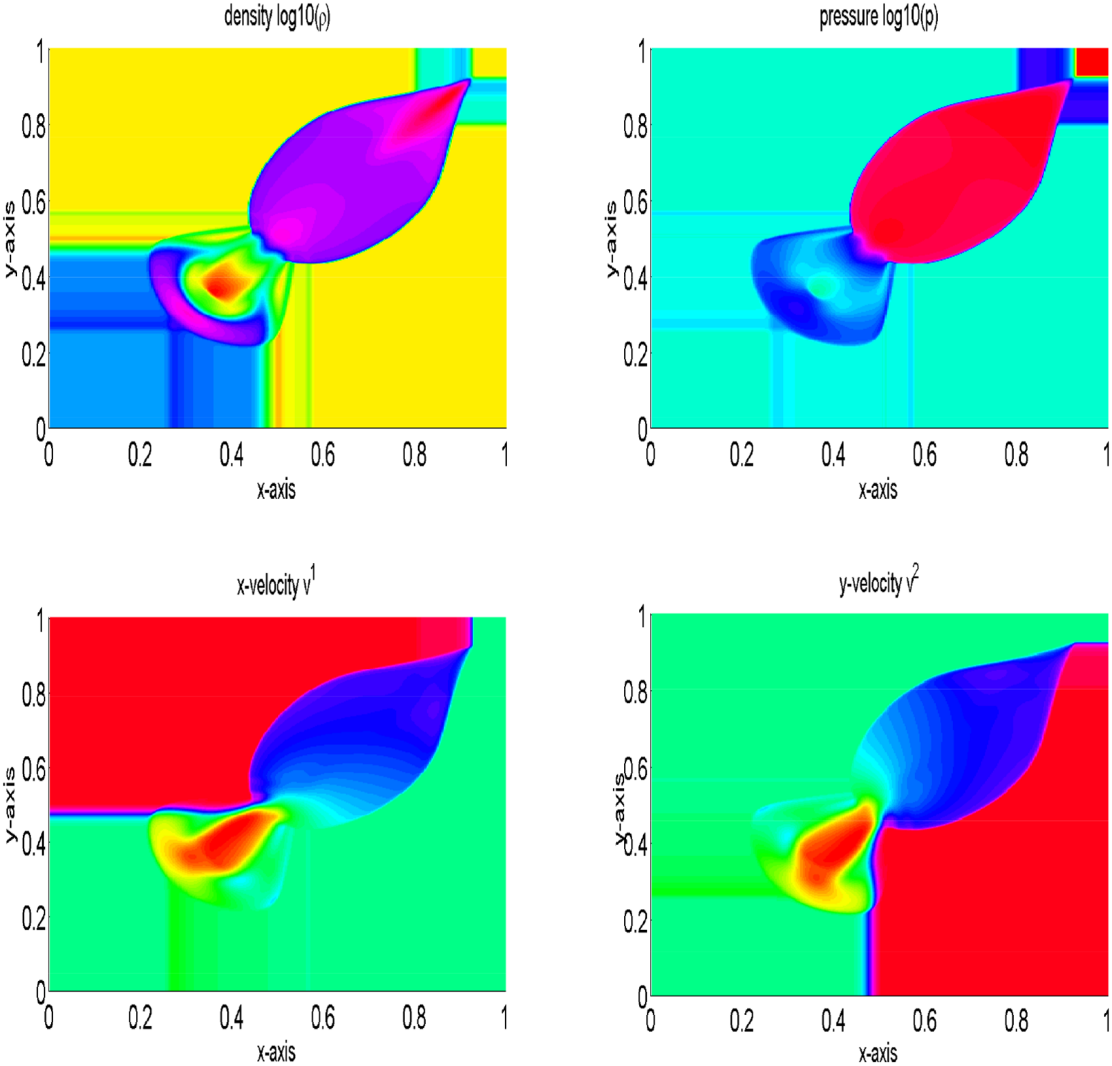
Results of two dimensional shock tube problem at time *t* = 0.4.

**Fig 6:**
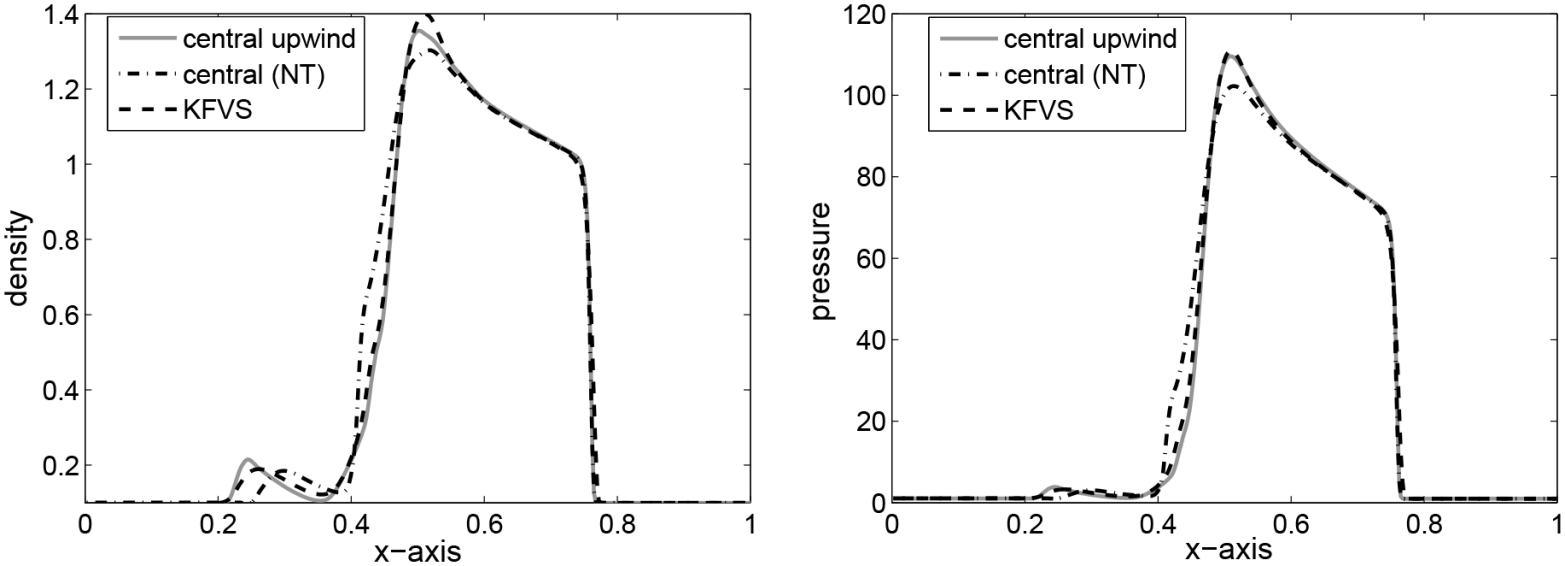
Comparison of results along x-axis at *y* = 0.5.

The periphery of quadrants specify two 1D shocks and two contact discontinuities. These are symmetric with respect to the main diagonal. It is to be noted that we have not considered an exact 1D shock across the N and E interfaces, and this may be identified by investigating the emerged discontinuities in Fig 5, focusing toward the NE direction with their complete Riemann fan. In the remanning domain, the structure emerges with curved shock fronts and a complex pattern in the SW quadrant, reminiscent of an oblique jet with converging internal shock fronts and a bow shock. The lines in the SW corner with respect to the bow shock are actually due to spurious waves produced at the S and W interfaces by the numerical diffusion term appearing in the energy equation, right and left states have jump in kinetic energy and cannot be eliminated even with a Roe-type solver [4]. The results are presented in Fig 5. Our results are identical to those available in [4]. Finally, Fig 6 gives a comparison of density and pressure obtained from the central upwind and the central(NT) schemes. It can be observed that the central upwind and KFVS schemes give more resolved solutions as compared to the central (NT) scheme.

#### Problem 7: Diffracting shock waves and the forward facing step

**Fig 7:**
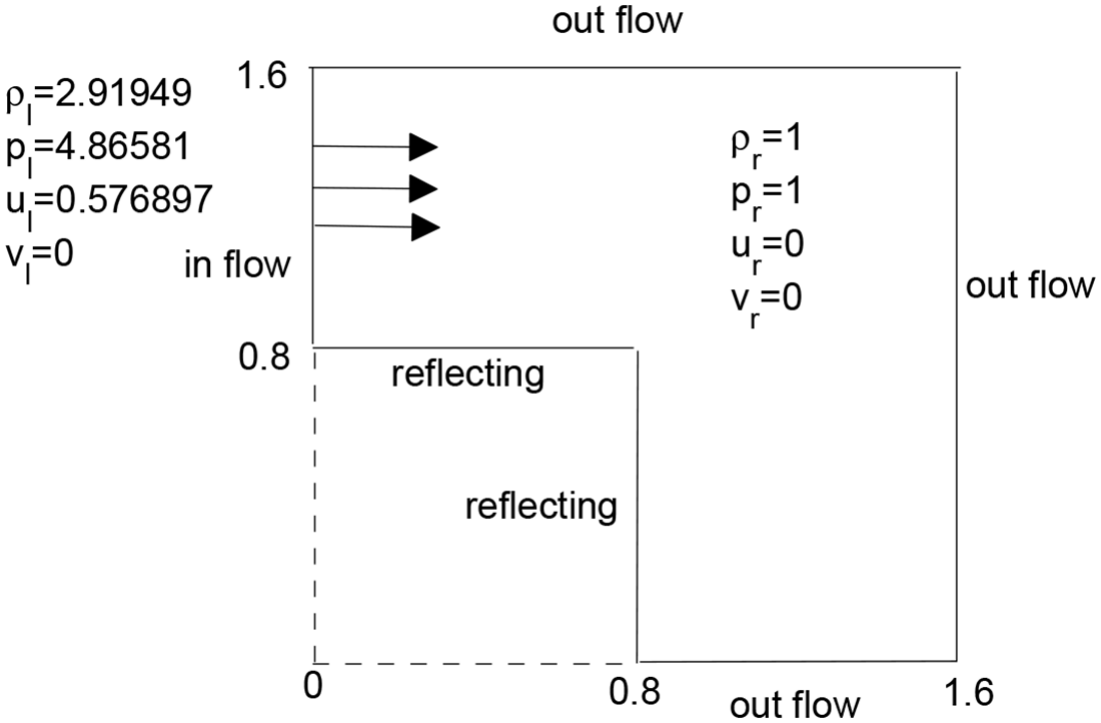
Initial data.

**Fig 8:**
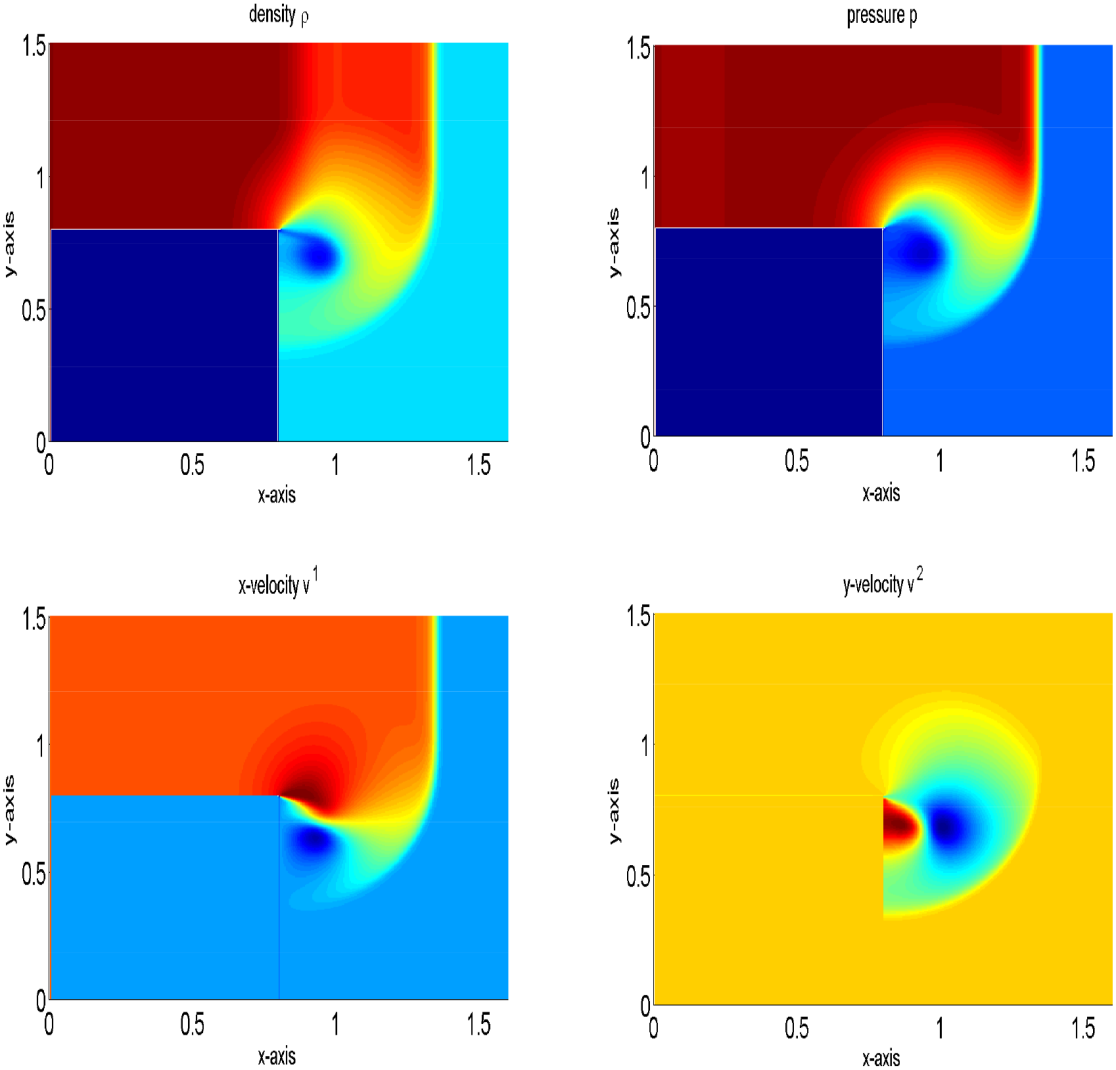
Results of diffracting shock waves and the forward facing step problem at time *t* = 1.7.

**Fig 9:**
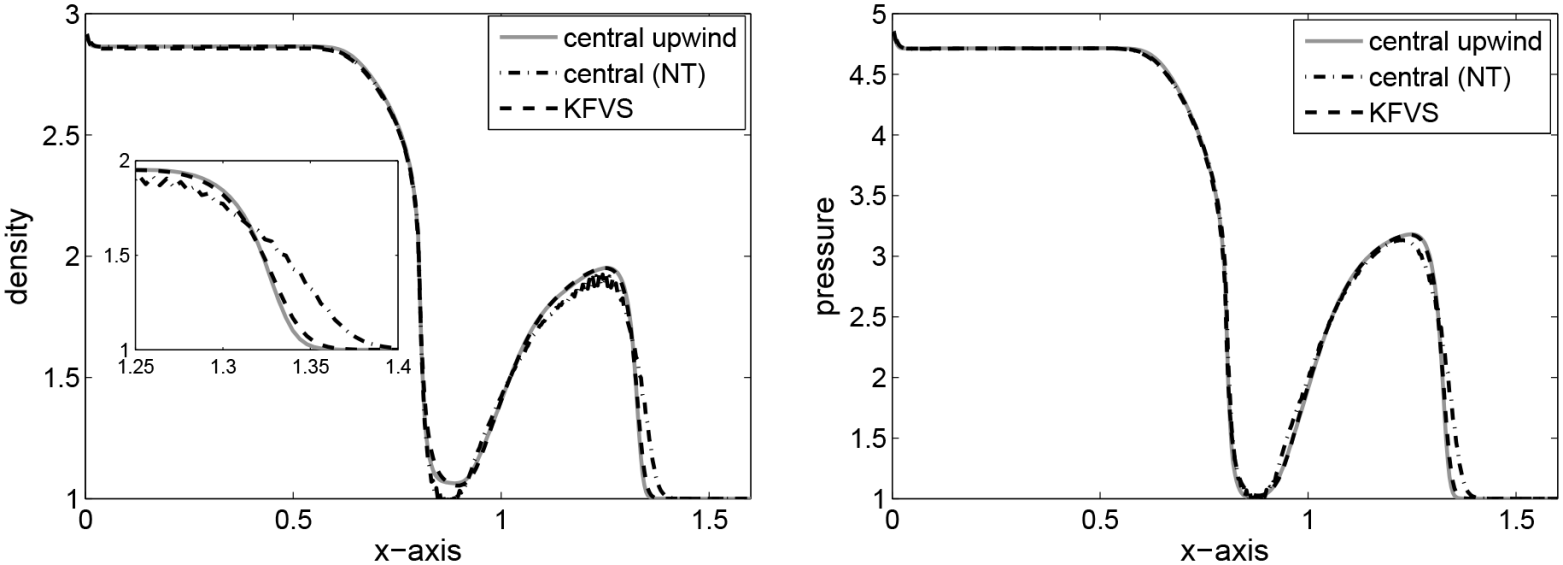
Comparison of results along x-axis at *y* = 0.5.

**Fig 10:**
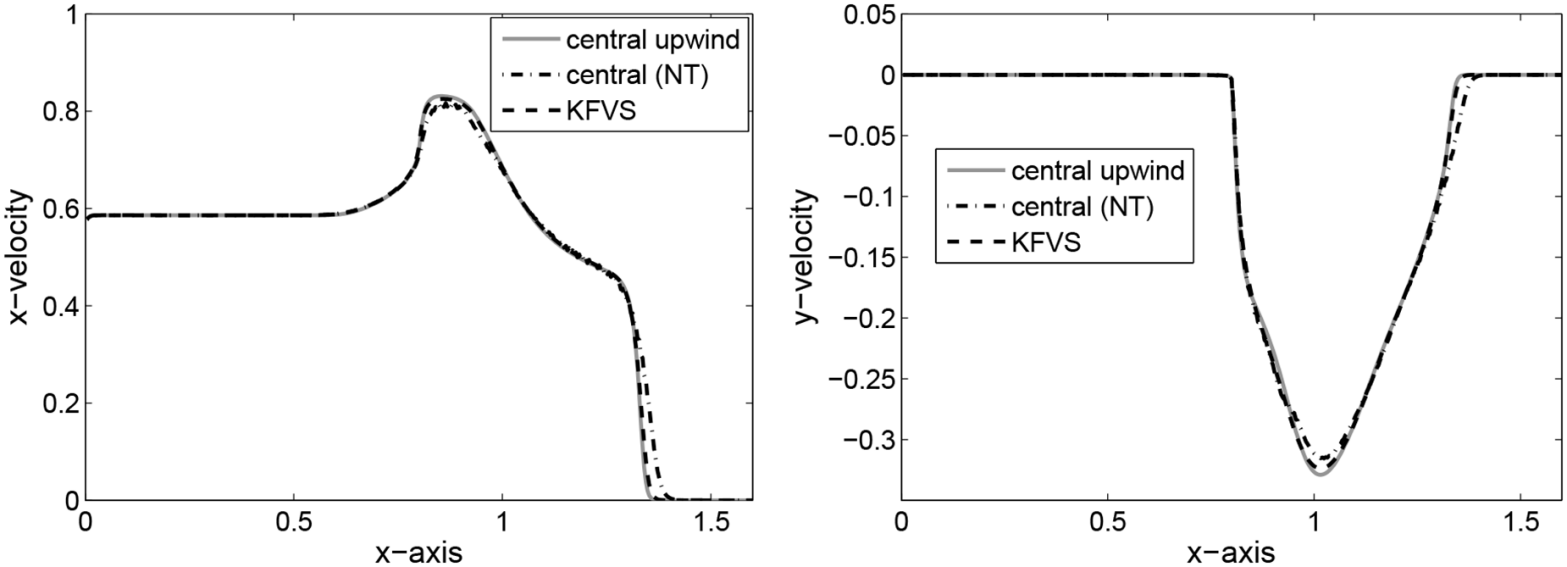
Comparison of results along x-axis at *y* = 0.5.

In this problem, the shock diffraction at a corner is considered. This is a relativistic analog of the classical experimental results presented by van Dyke [27]. Here, the dimensions of the square region are less than those presented in [27]. The initial conditions are given in Fig 7. The results are presented in Fig 8. There is a shock wave placed at *x* = 0 and a rolled-up vortex is produced at the corner. A grid of 300 × 300 points is taken and the total time for simulation is 1.6. Finally, Figs 9 and 10 give a comparison of the central upwind, KFVS and central(NT) schemes. It can be observed that the central upwind and KFVS schemes gives more resolved solutions as compared to the central (NT) scheme. Moreover, the density plots of central (NT) scheme are oscillatory.

#### Problem 8: Cylindrical Explosion Problem

**Fig 11:**
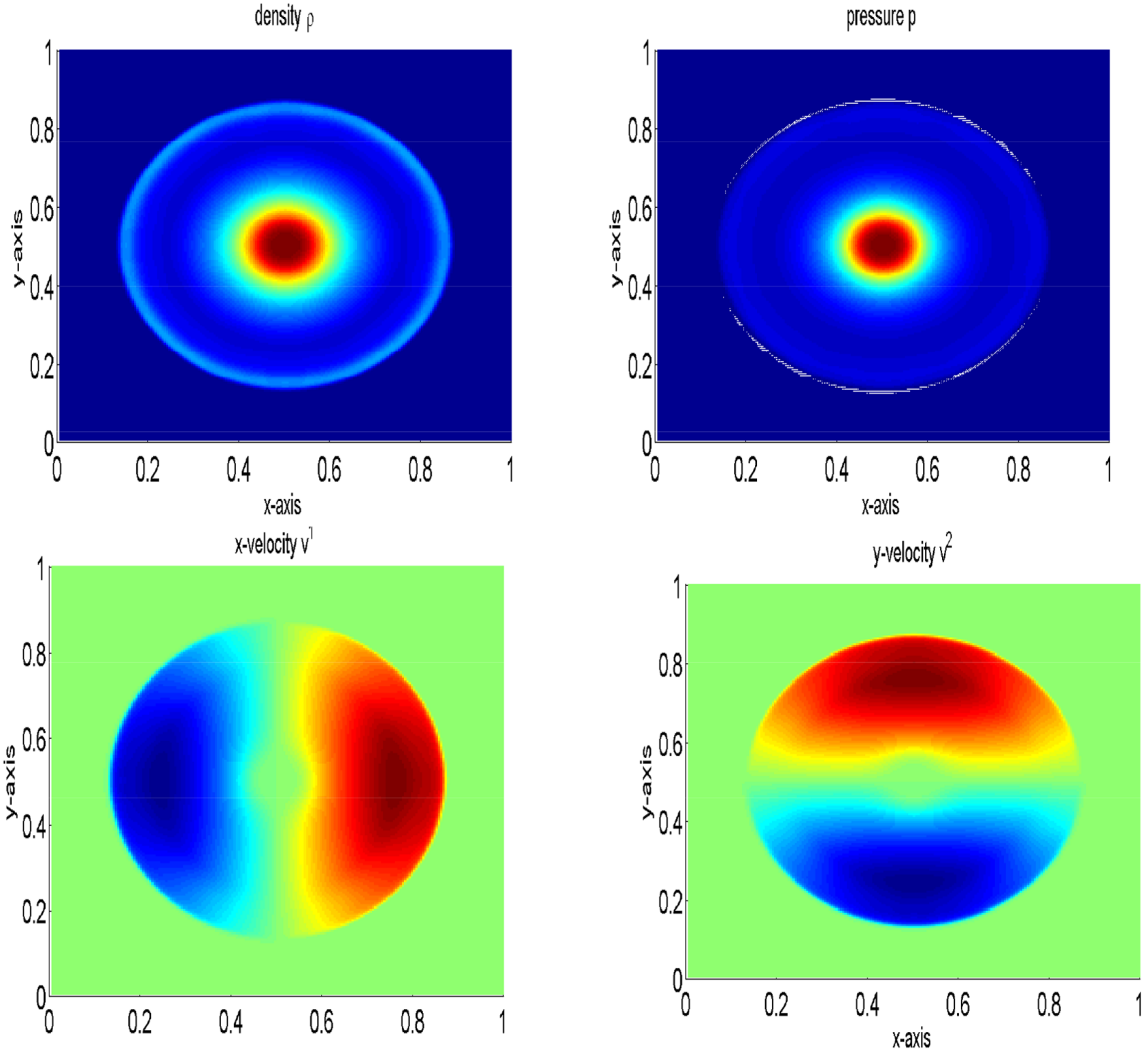
Comparison of the schemes on 200 × 200 grid points at *t* = 0.2.

**Fig 12:**
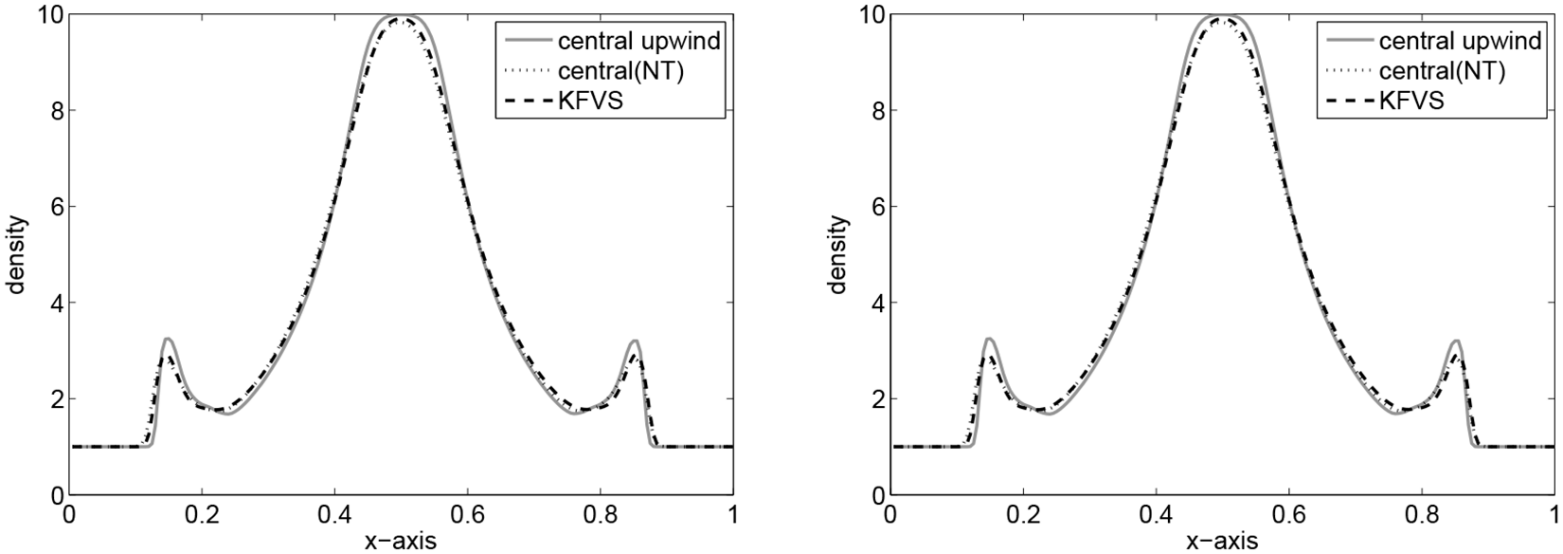
Comparison of results along x-axis at *y* = 0.5.

Here, a two-dimensional problem is examined. We consider a squared domain [0, 1] × [0, 1] which is divided into 200 × 200 mesh points. The initial data are constant in two regions separated by a circle of radius 0.2 with center (1/2, 1/2). Inside the circle *ρ* is 10.0 and *p* is 10, while outside the circle *ρ* is 1 and *p* is 0.01. The velocities are zero everywhere. In the solution, a circular shock wave propagates outwards from the origin, followed by circular contact discontinuity traveling in the same direction, and a circular rarefaction wave propagating towards the origin. Fig 11 shows the results of the central upwind scheme at final time t = 0.2. In Fig 12, the results of the central upwind scheme are compared with the central(NT) and KFVS schemes results. It can be observed that the results of all schemes agreeing well. However, the central upwind scheme has resolved solution than other two schemes.

#### Problem 9: Explosion in a box

**Fig 13:**
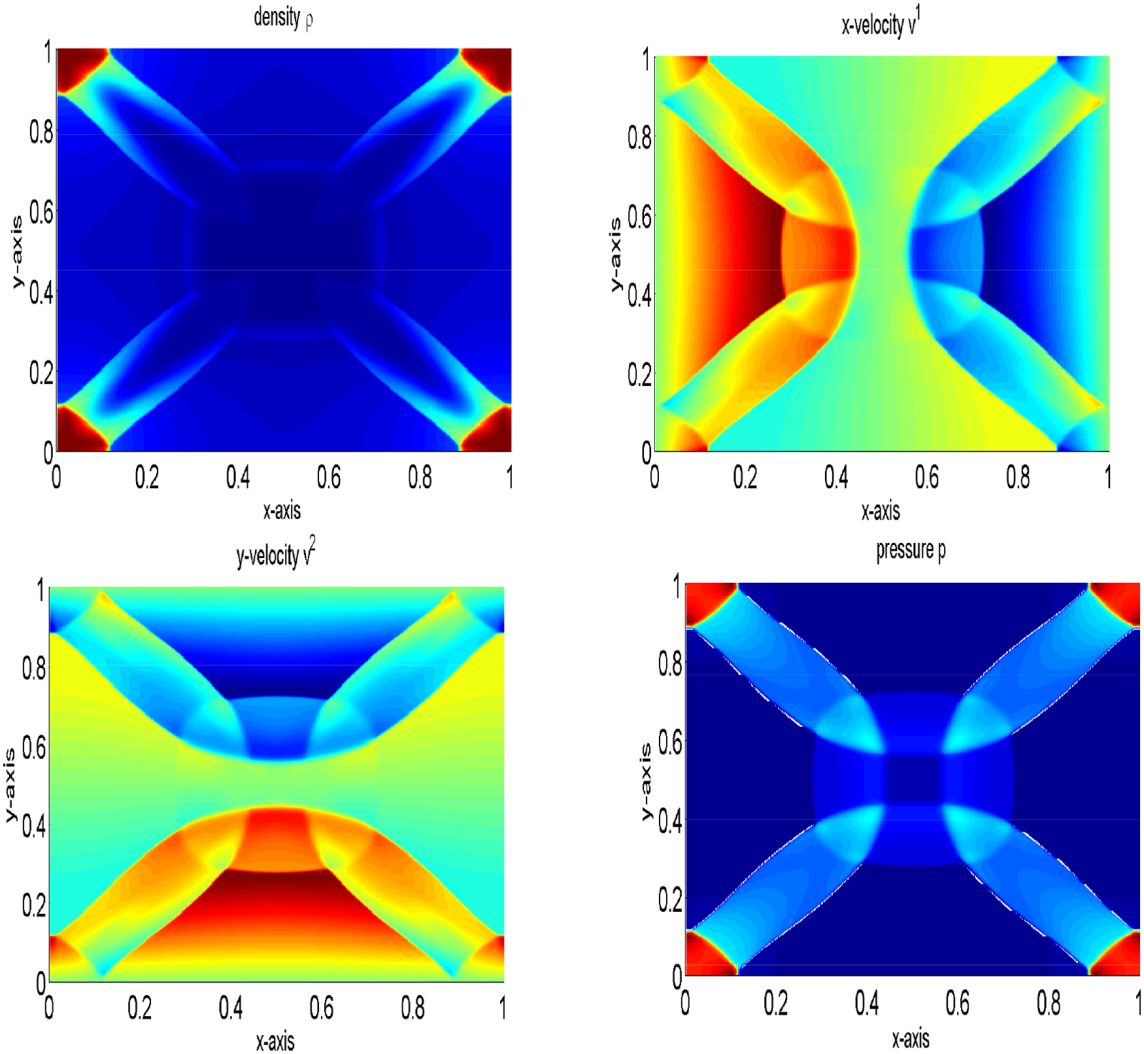
Results of explosion in a box at time *t* = 1.0.

**Fig 14:**
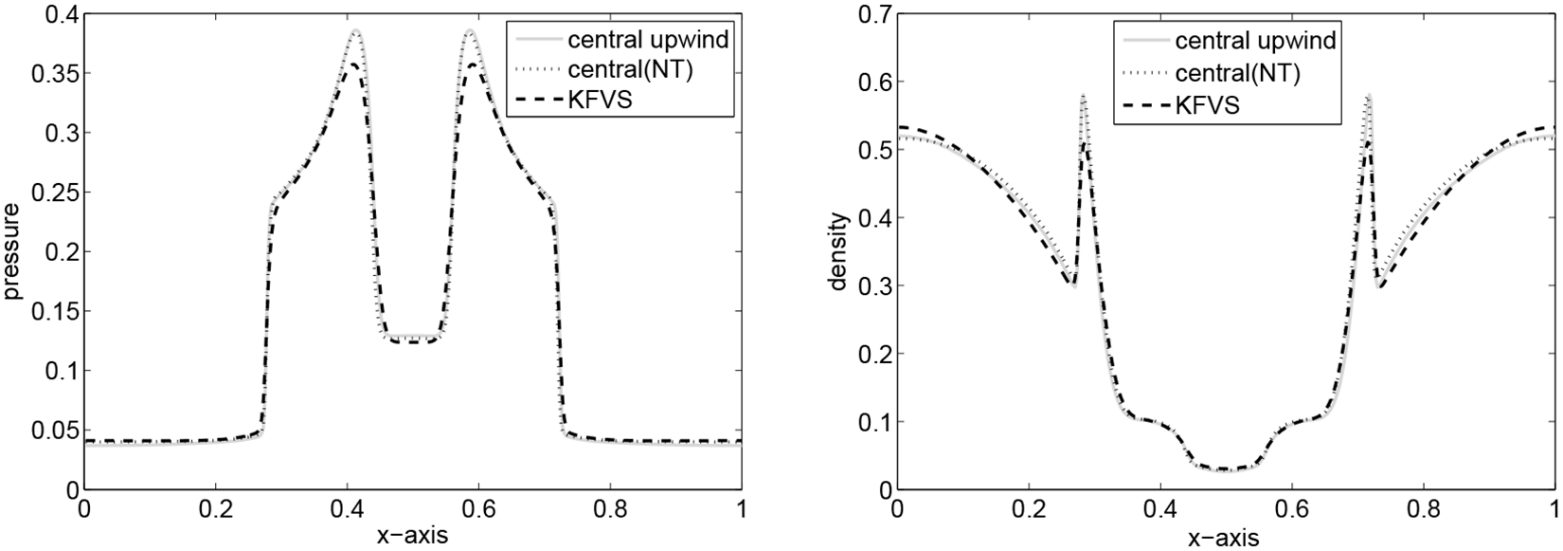
Comparison of results along x-axis at *y* = 0.5.

**Fig 15:**
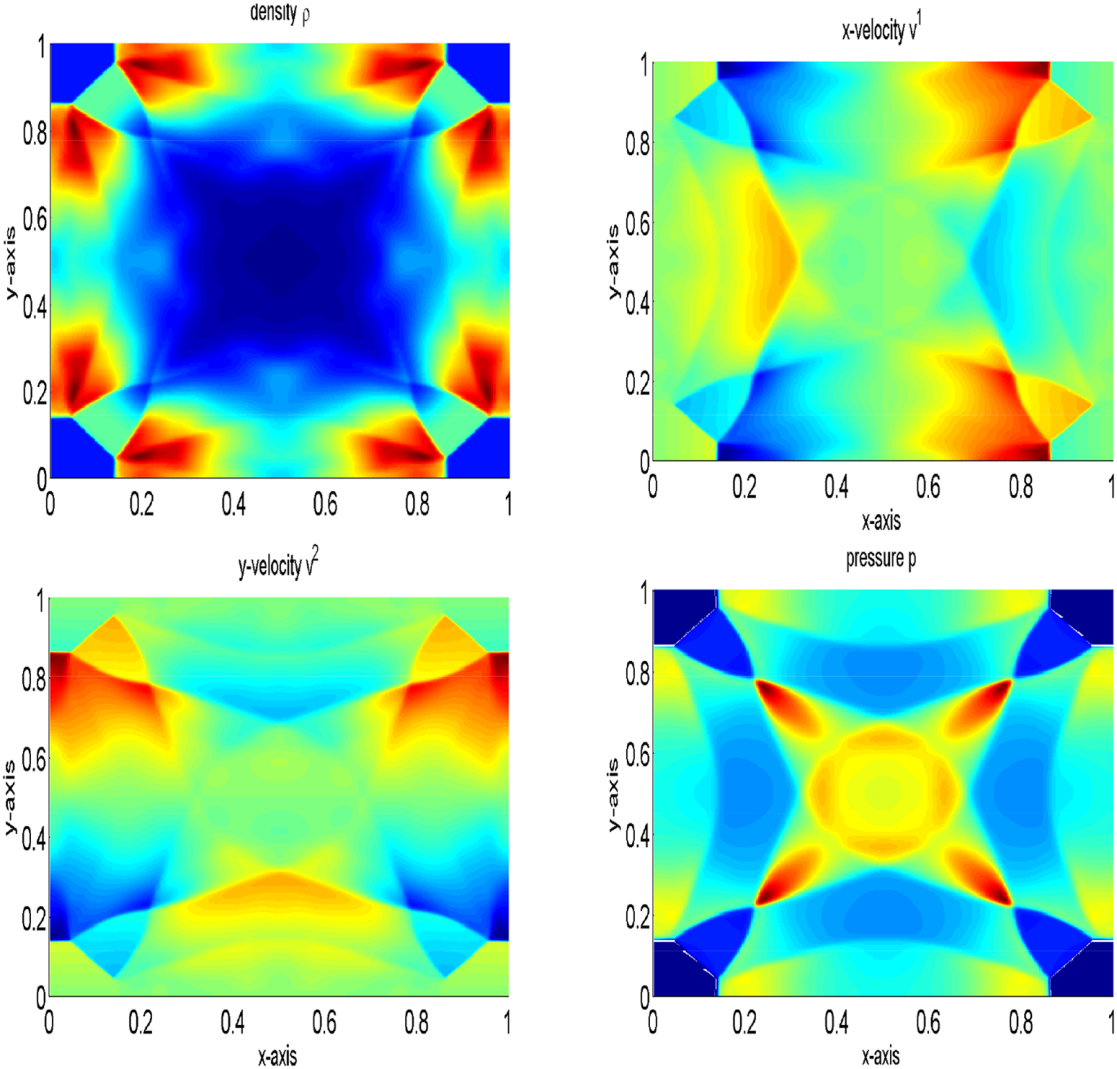
Results of explosion in a box at time *t* = 2.0.

**Fig 16:**
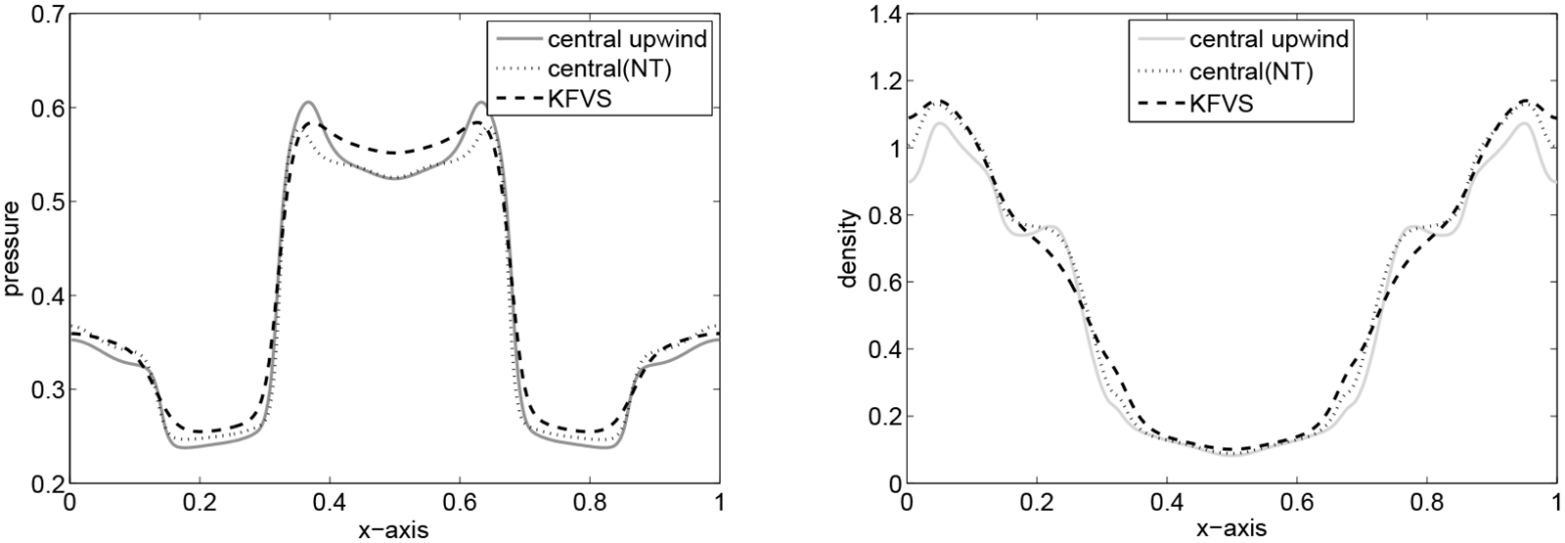
Comparison of results along x-axis at *y* = 0.5.

This test problem corresponds to a 2D Riemann problem within a square box having unit side length. The walls are reflecting. The initial velocities are zero, while unit density is taken. The pressure is 10 inside a small square box of sides length 0.2 in the middle of the large box, while it is 0.01 elsewhere. The computational domain is discretized into 400 × 400 mesh points. The numerical results at *t* = 1.0 and *t* = 2.0 are shown in Figs 13–16, respectively. In Figs 14 and 16 the numerical results of the central upwind scheme are compared with the KFVS and central(NT) schemes. It was found that all schemes give equivalent results. However, central scheme seems to be more superior.

## 4 Conclusions

In this article, the central upwind scheme was applied to solve special relativistic Euler equations in one and two space dimensions. The suggested method has capability to accurately capture the discontinues profiles of relativistic fluid and avoids numerical diffusion and dispersion in the solution. It was found that suggested scheme is second order accurate for smooth initial data in one and two space dimensions. The numerical errors in the solutions are less for the central upwind scheme and are larger for the central (NT) scheme. In most of the considered problems, the numerical results of KFVS and central upwind schemes are comparable and central (NT) scheme produced diffusive results. However, in some problems, the results of central upwind scheme were found to be superior than the other two schemes. It was found that the computational costs of all schemes were of the order of few seconds in the one-dimensional case and of the order few minutes in the two-dimensional case.
